# Lack of detection of a human placenta microbiome in samples from preterm and term deliveries

**DOI:** 10.1186/s40168-018-0575-4

**Published:** 2018-10-30

**Authors:** Jacob S. Leiby, Kevin McCormick, Scott Sherrill-Mix, Erik L. Clarke, Lyanna R. Kessler, Louis J. Taylor, Casey E. Hofstaedter, Aoife M. Roche, Lisa M. Mattei, Kyle Bittinger, Michal A. Elovitz, Rita Leite, Samuel Parry, Frederic D. Bushman

**Affiliations:** 1 Department of Microbiology, University of Pennsylvania School of Medicine, 3610 Hamilton Walk, Philadelphia, PA 19104-6076 USA; 2 Division of Gastroenterology, Hepatology, and Nutrition, The Children’s Hospital of Philadelphia, Philadelphia, PA 19104 USA; 3 Maternal and Child Health Research Center, Department of Obstetrics and Gynecology, University of Pennsylvania School of Medicine, 3400 Spruce Street, Philadelphia, PA 19104 USA

**Keywords:** Placenta, Shotgun metagenomics, 16S rRNA gene, Microbiome, Preterm birth

## Abstract

**Background:**

Historically, the human womb has been thought to be sterile in healthy pregnancies, but this idea has been challenged by recent studies using DNA sequence-based methods, which have suggested that the womb is colonized with bacteria. For example, analysis of DNA from placenta samples yielded small proportions of microbial sequences which were proposed to represent normal bacterial colonization. However, an analysis by our group showed no distinction between background negative controls and placenta samples. Also supporting the idea that the womb is sterile is the observation that germ-free mammals can be generated by sterile delivery of neonates into a sterile isolator, after which neonates remain germ-free, which would seem to provide strong data in support of sterility of the womb.

**Results:**

To probe this further and to investigate possible placental colonization associated with spontaneous preterm birth, we carried out another study comparing microbiota in placenta samples from 20 term and 20 spontaneous preterm deliveries. Both 16S rRNA marker gene sequencing and shotgun metagenomic sequencing were used to characterize placenta and control samples. We first quantified absolute amounts of bacterial 16S rRNA gene sequences using 16S rRNA gene quantitative PCR (qPCR). As in our previous study, levels were found to be low in the placenta samples and indistinguishable from negative controls. Analysis by DNA sequencing did not yield a placenta microbiome distinct from negative controls, either using marker gene sequencing as in our previous work, or with shotgun metagenomic sequencing. Several types of artifacts, including erroneous read classifications and barcode misattribution, needed to be identified and removed from the data to clarify this point.

**Conclusions:**

Our findings do not support the existence of a consistent placental microbiome, in either placenta from term deliveries or spontaneous preterm births.

**Electronic supplementary material:**

The online version of this article (10.1186/s40168-018-0575-4) contains supplementary material, which is available to authorized users.

## Background

Infection of the placenta by pathogens is known to cause adverse outcomes in pregnancy [1, 2]. Historically, the contents within the gravid uterus of a healthy pregnancy were believed to be sterile based on numerous attempts to culture living microorganisms from placental tissue and amniotic fluid [3–5]. In contrast, in recent years, it has been proposed that microorganisms colonize the placenta naturally based on deep DNA sequencing data, so that a placenta microbiome is characteristic of health [6–13]. In one study, lineages found in placenta samples were proposed to most closely match lineages from oral samples [6]. Imaging studies also reported possible identification of intracellular bacteria within the basal plate of placentas and at the maternal-fetal interface [14].

Previously, we reported that we could not detect a placenta microbiome over the background in negative controls in samples from term deliveries [15]. In this previous study, analysis of the absolute quantities of bacterial 16S rRNA gene copies using qPCR showed no difference between the amount of DNA extracted from placental tissues versus background negative controls. Sequencing 16S rRNA marker genes showed that bacterial lineages present in placenta samples and negative controls were indistinguishable, but differed from oral or vaginal swab samples run in parallel, which showed specific lineages typical of each anatomical site. Several groups have published on contaminants present in extraction reagents and the complications this introduces in studying samples with low microbial biomass [16–18], such as samples of placenta tissue.

Another type of data argues strongly for sterility within the amniotic sac in healthy pregnancies. For many mammals, it is possible to create axenic (germ-free) neonates by delivery using sterile cesarean section, followed by introduction of the neonate into a sterile isolator. This method is used routinely to derive germ-free mouse strains and has been used to derive germ-free rats, guinea pigs, rabbits, dogs, cats, pigs, lambs, calves, goats, baboons, chimpanzees, and marmosets [19]. Even germ-free humans have been generated by this method [19, 20]. This observation provides strong evidence against the idea that contents of the gravid uterus (placenta, amniotic cavity, and fetus) are colonized with microbes.

Here, we sought to investigate possible placenta colonization associated with spontaneous preterm birth. We reasoned that even if the healthy placenta is not normally colonized, perhaps placenta associated with preterm delivery would harbor a detectable microbiome. We thus obtained placenta tissue and control samples from 20 term and 20 spontaneous preterm births (Table 1). We first carried out 16S rRNA gene qPCR to check absolute levels of bacterial DNA, but found that both term and preterm samples had low copy numbers indistinguishable from background levels in negative controls. We then carried out both 16S rRNA gene sequencing and shotgun metagenomic sequencing, but found no consistent microbial signature unique to placenta in term or preterm births.

**Table 1:** Summary of human subjects studied

Characteristic	Term (*N* = 20)	Preterm (*N* = 20)
Maternal age (year)	26.5 (20–34)	27.6 (18–44)
Race (%)		
AA	55	55
White	30	40
Asian	10	0
Other	5	5
Nulliparous (%)	50	40
Mode of delivery (%)		
SVD	95	75
C-section	5	25
(P)PROM (%)	40	65
Intrapartum Abx (%)	20	85

Detailed metadata on each individual is in Additional file 1: Table S1

*AA* African American, *SVD* spontaneous vaginal delivery, *(P)PROM* (preterm) premature rupture of the membranes

## Results

### Birth cohorts studied

To investigate possible colonization of the placenta associated with preterm birth, we analyzed 20 placentas from spontaneous preterm births and 20 from full-term deliveries (Table 1 and Additional file 1: Table S1). Placenta tissue samples were processed to remove the external layers in an effort to exclude microbes adhering to the outside of the placenta acquired during delivery. Maternal side and fetal side samples were both collected. Six cases of preterm birth were complicated by both clinical chorioamnionitis (maternal fever > 100.4 and at least one of the following: maternal tachycardia > 100 bpm, fetal tachycardia > 160 bpm, or fundal tenderness) and histological chorioamnionitis (white blood cells detected in fetal membranes). As positive controls, we collected saliva and cervicovaginal fluid from the mothers—both body sites that are known to harbor rich microbial communities [21–23]. We also collected several types of negative controls (Additional file 1: Table S2). These included (1) swabs that were opened in the sample processing room and waved in the air (“Air Swab”), (2) empty tubes that were processed through the DNA extraction (“Blank”), and (3) PCR grade water processed in parallel with the samples during amplification and DNA sequencing acquisition, though not DNA purification (“H2O”).

### DNA purification

In our previous work, we used two different DNA purification kits to prepare samples, because purification kits are known to be sources of contaminating bacterial DNA [16, 17]. Previously, we used the MO BIO PowerSoil and PSP kits and found that each kit yielded DNA producing distinct bacterial sequences when amplifying negative controls. DNA extracted from placenta samples with the two kits not only resembled negative controls but also matched the particular background of each extraction kit, helping us distinguish the influence of reagent contamination. Thus, in this study, we also used two DNA purification kits (Additional file 1: Table S3). We used the DNeasy PowerSoil kit to match previous work [6, 15], and also, in an attempt to suppress the influence of contamination, we compared a newly available DNA purification kit designed to minimize adventitious DNA, the QIAamp UCP Pathogen Mini kit (UltraClean). As positive controls, we also purified samples expected to contain robust microbial communities from maternal saliva and vaginal swabs. Only one set of vaginal swabs was available, so these were purified using the UltraClean kit.

### Absolute abundance of bacterial DNA in samples quantified using 16S rRNA gene-targeted qPCR

**Fig. 1:**
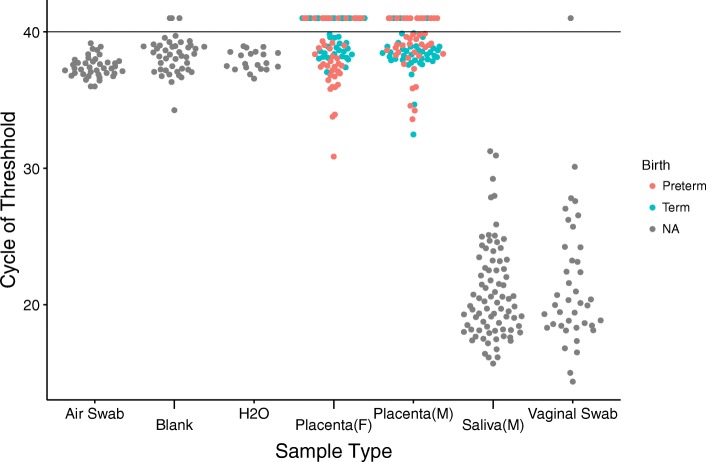
Quantitative PCR (qPCR) analysis of the 16S rRNA gene abundance in the samples studied. Values shown are the cycle of threshold (CT) of each sample. The limit of detection is a CT level of 40 (horizontal line). Samples with no detectable signal are shown above the line. Statistical comparisons of between sample types are in Additional file 1: Table S4. Data was pooled for samples generated using two DNA different extraction kits

We investigated our sample set to determine whether placenta DNA samples contained higher absolute levels of bacterial DNA than negative controls as measured using 16S rRNA gene qPCR (Fig. 1). Samples from saliva and cervicovaginal fluid contained high concentrations of bacterial DNA (low cycle of threshold), as expected (Fig. 1, right two sample sets). Negative controls showed high cycles of threshold, indicating low 16S rRNA gene content (Fig. 1, leftmost three sample sets). Placenta samples contained little 16S rRNA gene DNA, with cycles of threshold similar to negative controls. (Fig. 1, middle). Comparisons between the placenta samples and Blank and H2O samples show that there was no significant difference (Placenta (F)–Blank, *p* = 0.915; Placenta (F)–H2O, *p* = 0.392; Placenta (M)–Blank, *p* = 0.958; Placenta (M)–H2O, *p* = 0.426; Kruskal-Wallis with Dunn’s post-test) (Additional file 1: Table S4). We found significant differences between placenta samples and Air Swabs (*p* = 0.001 for fetal side, *p* = 0.002 for maternal side); however, the negative control Air Swabs yielded a lower CT (higher amounts of bacteria; Additional file 1: Table S4). We found no differences between sample sets when comparing placenta samples separated by term, chorioamnionitis, or extraction kit with the negative controls (Additional file 1: Tables S5-S10).

We then compared placenta samples between term and preterm births. Two comparisons achieved significance, but the direction was inconsistent. For data from the UltraClean kit, comparison of fetal side placenta from preterm and term births showed a significantly lower mean value CT for the preterm births (*p* < 10^−5^, Kruskal-Wallis). However, for data from the PowerSoil kit, comparing the maternal side placenta data to controls showed lower mean CT for the term births (*p* = 0.0396, Kruskal-Wallis). We compared the chorioamnionitis placenta samples to other preterm birth and pooled placenta samples and found no significant differences. We conclude that, unlike the saliva or vaginal samples, the numbers of 16S rRNA gene copies in placental samples were not significantly higher than in negative controls and that there is little difference between preterm, term or chorioamnionitis samples.

### Analysis of bacterial DNA using 16S rRNA marker gene sequencing

We next assessed the composition of the 16S rRNA gene sequences in our samples using 16S rRNA marker gene sequencing. For this, we analyzed the V1-V2 region of the 16S rRNA gene, because previous work suggested that the placental microbiome might be derived from oral communities [6], and V1-V2 has been used to characterize oral communities in multiple peer-reviewed publications [24–29]. This region of the gene is also relatively short (typically ~ 260 bp), which increases amplification efficiency and so is useful for analyzing low biomass samples [24, 26]. We amplified and sequenced the sample set (Additional file 1: Table S3) and as an additional positive control added a set of synthetic DNAs encoding divergent microbial 16S rRNA gene sequences to verify robust performance of biochemical steps and proper sample tracking [17]. We recovered useable data (> 100 reads) for 303 of 388 samples (Additional file 1: Table S11 and Additional file 2: Figure S1). Of the placenta samples, 62/160 (38.75%) did not meet this threshold. We speculate that clean work up of low biomass samples resulted in suppression of background DNA contamination, leading to fewer 16S rRNA sequencing reads in some samples. The output 16S rRNA gene sequences were clustered using Dada2, and clusters were assigned taxonomically using the SILVA database.

**Fig. 2:**
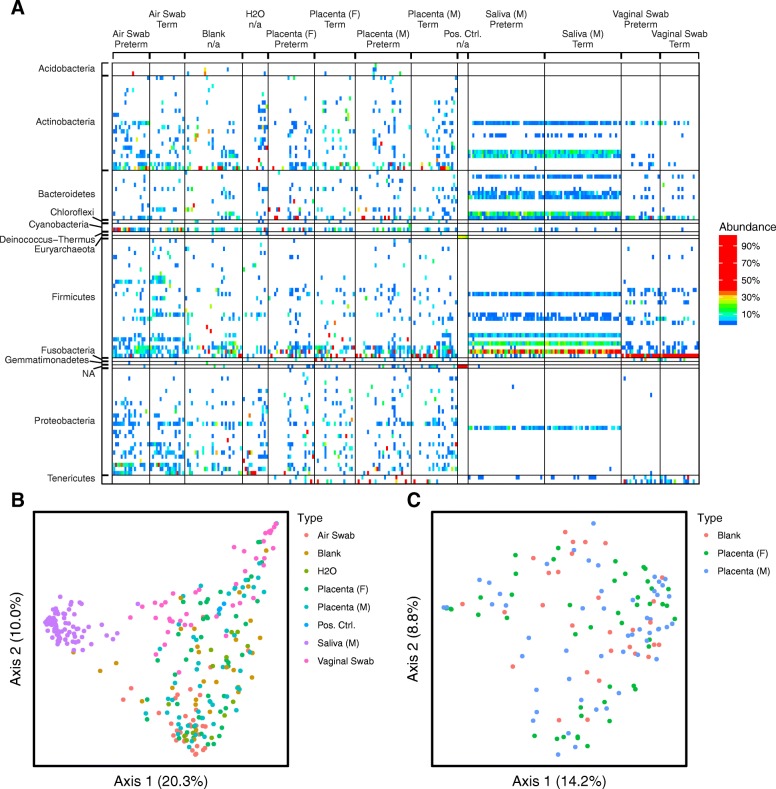
Overview of bacterial abundances inferred using 16S rRNA marker gene sequencing. **a** Heatmap showing relative abundances of bacterial taxa per sample (columns). Reads were aggregated at the genus level, which are shown as the different rows. Denser sample labeling is available in Additional file 2: Figure S2. Samples are grouped by sample type and further grouped by term or preterm delivery (more detailed sample labeling in Additional file 2: Figure S2). **b** PCoA of unweighted UniFrac distances, for all samples, colored by sample type. *p* values for group comparisons are in Additional file 1: Table S12. **c** PCoA of unweighted UniFrac comparing blanks to placenta samples

The major lineages detected are summarized in Fig. 2a and detailed further in Additional file 2: Figure S2. Vaginal and oral samples showed well-known bacterial lineages associated with these body sites; vaginal samples contained high proportions of Ureaplasma, Sneathia, and Lactobacillus, and oral samples were high in Neisseria, Porphyromonas, Streptococcus, and Prevotella [21–23]. Samples from placenta, and also the negative controls, were high in environmental bacteria commonly associated with contaminants, such as Ralstonia and Pseudomonas[16]. Placenta and negative control samples also contained reads matching to chloroplasts (Streptophyta), possibly derived from pollen in dust. Other bacteria present in both placenta and negative control samples included Prevotella and Enterobacteraceae, which may be either human-associated or environmental. Several vaginal lineages were detected selectively in some of the placenta samples, including Ureaplasma, Sneathia, and Lactobacillus. Further analysis showed that these were found predominantly in the samples from vaginal deliveries and not cesarean deliveries. To test this further, we asked whether vaginal lineages found in placenta matched between mother-placenta pairs more closely than in unmatched mother-placenta pairs; placentas were found to share lineages with their corresponding mother more frequently than with other mothers (*p* < 10^−5^ for comparison of Jaccard distances). In contrast, samples from cesarean deliveries showed no such correspondence (*p* = 0.24 for comparison of Jaccard distances), supporting the idea that vaginal lineages in placentas were derived from the mother during vaginal delivery.

Clustering of samples using unweighted UniFrac showed separation of the saliva and vaginal samples from all other sample types, while negative controls and placenta samples formed an overlapping large cluster (Fig. 2b). Sample clustering was then assessed statistically by PERMANOVA (all *p* values are in Additional file 1: Table S12), comparing weighted UniFrac, unweighted UniFrac, and Jaccard distances.

Comparison of placenta samples from preterm and term deliveries showed no significant difference. Comparison of vaginal delivery versus cesarean delivery also showed no difference for the UniFrac analyses, but did achieve significance for Jaccard distance. Distances were significantly different between kits (*p* = 0.021 all samples, fetal, maternal). Comparisons between preterm and term deliveries, performed separately on samples of each kit, showed no differences. Comparisons of vaginal delivery versus cesarean delivery, performed separately on samples purified using each kit, also showed no difference, suggesting that there are no strong distinctions based on delivery type in the data.

Comparisons of sample type showed significant differences between blanks versus saliva, vaginal, and air swab samples. Significant differences were also found when comparing samples of each kit. However, blanks and placenta samples showed no difference between sample type (Fig. 2c). Comparisons of blanks with placenta samples performed on samples from each kit separately also did not show significant differences.

Comparison of placenta samples associated with chorioamnionitis showed no significant difference compared to other placenta samples. An overview of the bacterial lineages detected is in Additional file 2: Figure S3. Recently, Leon et al. [30] published a study of 265 pregnancies, assessing the possible placenta microbiome in term and preterm samples using 16S rRNA amplicon sequencing. They too found no consistent placenta microbiome over background. They did however find representation of Ureaplasma and Mycoplasma in some preterm placentas. In our data, we assessed possible colonization with these organisms, requiring detection by 16S rRNA amplicon sequencing in at least 2/4 samples per placenta to call a lineage as present. Analysis was carried out at the level of operational taxonomic units, our most discriminating measure (Additional file 2: Figure S4). We found Mycoplasma in 2/20 preterm deliveries and none of the term deliveries; we found Ureaplasma in 3/20 preterm deliveries and 1/20 term deliveries. Thus, although there was no statistically significant difference between our preterm and term samples, detections of Mycoplasma and Ureaplasma in the 16S rRNA amplicon data from preterm samples are potentially consistent with results of Leon et al. However, we note that 5/6 cases are in vaginally delivered placentas and the organism could be detected in the vaginal sample from the same woman (Additional file 2: Figure S4). Thus, contamination with vaginal microbes during delivery is also a possible explanation for the origin of Ureaplasma and Mycoplasma in the preterm placenta samples.

The exception was a unique Ureaplasma amplicon sequence variant detected in two placenta samples and not in the corresponding vaginal swab from a preterm, cesarean section, and chorioamnionitis-associated birth (subject 265). Ureaplasma has been associated with chorioamnionitis, and thus in this case may represent detection of an authentic infection in subject 265 [31], paralleling results of Leon et al. [30].

We conjecture that kit contamination was a less significant factor here than in Lauder et al. [15] and that sequences from other sources, such as laboratory water (e.g., Ralstonia) and maternal vagina were the major source of contamination in this study. Thus, our analysis of 16S rRNA marker gene sequencing did not disclose any consistent distinction between placenta samples and negative controls.

### Analysis of total DNA content using shotgun metagenomic sequencing

Previous studies have used shotgun metagenomic sequencing to analyze the placenta microbiome [6], so we decided to also compare shotgun sequencing for this sample set. A major disadvantage of using shotgun metagenomics to interrogate human tissue is that the vast majority of sequencing effort will be spent on sequencing human DNA. We thus focused on sequencing a subset of our samples, so as to obtain more sequencing depth. We chose to sequence only the samples extracted with the UltraClean kit because that set included the vaginal swabs.

**Fig. 3:**
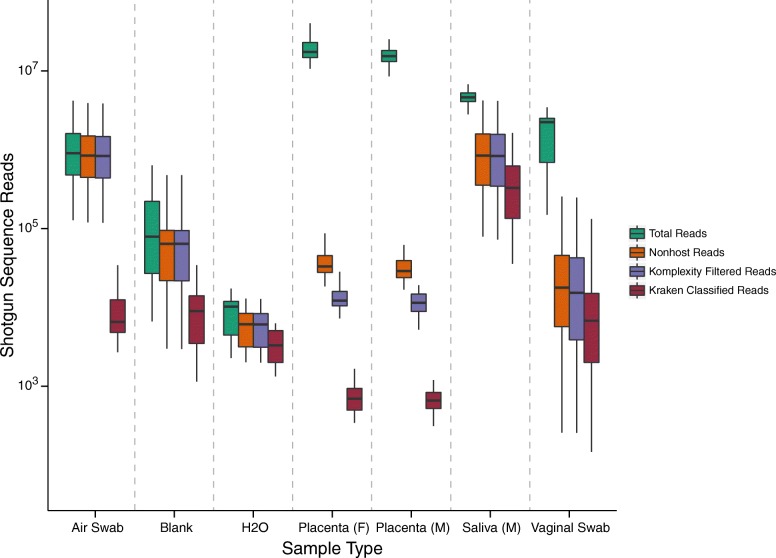
Reads counts from shotgun metagenomic sequence analysis separated by sample type. “Total Reads” are all reads from the HiSeq sequencing run; “Nonhost Reads” are the reads remaining after Sunbeam human filtering, “Komplexity Filtered Reads” are those remaining after filtering low-complexity reads from the Nonhost reads, “Kraken Classified Reads” are those that remained after Komplexity filtering and were classified with Kraken, after removing Chordata, Arthropoda, and Apicomplexa

Deep sequencing produced a large number of reads for each sample type: 1.4 billion reads for placenta, 31 million for negative controls, 190 million for saliva, and 72 million for vaginal samples (Fig. 3). Filtering out sequences matching the human genome removed large proportions of reads: 99.8% of the placenta samples, 19.3% of the negative controls, 76.0% of the saliva, and 97.8% from the vaginal swabs. The large proportion of human sequences removed from the placenta samples left only an average of 35,600 total reads per sample to interrogate for microbial composition.

Reads were then filtered a second time using Komplexity to mask low-complexity reads likely derived from host repetitive DNA elements such as microsatellites that eluded the first filter [32]. Next, the reads were assigned taxonomically using Kraken [33]. The reads were filtered a final time to remove those classified as Chordata and Arthropoda, which were likely human sequences that eluded earlier filtering steps, and Apicomplexa, unlikely placental colonists, because database genomes of this group are known to be problematic [34]. Finally, Kraken assignments with three or fewer classified reads (summed over the full sample set) were removed.

**Fig. 4:**
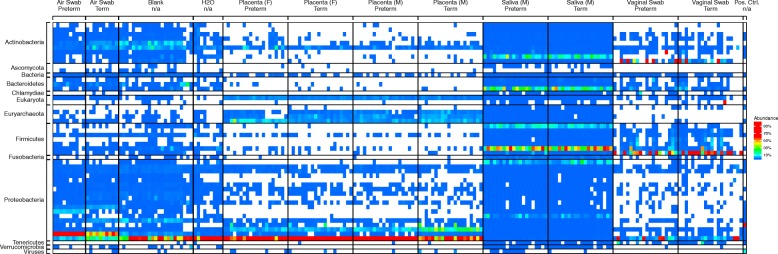
Heatmap showing the relative abundance of classified reads in each sample from shotgun metagenomic sequencing. Columns show individual samples, rows show genuses, grouped by phylum. The lineages shown are the top three most abundant classifications per sample. Denser sample labeling is available in Additional file 2: Figure S5

Major lineages detected for each sample are shown in Fig. 4 and detailed further in Additional file 2: Figure S5; placenta samples only are summarized in Additional file 2: Figure S6. Saliva samples showed the expected oral bacterial lineages, including Streptococcus and Prevotella. Vaginal lineages were dominated by Lactobacillus and Gardnerella. Placenta samples and the negative controls contained high proportions of Ralstonia, a bacteria known to be a frequent reagent contaminant [16], which was also detected in the 16S rRNA marker gene sequence data. Thus, the major placenta bacterial lineage in the shotgun sequencing data could be attributed to contamination.

The next two most abundant lineages in placenta, Alteromonas mediterranea and Methanosarcina mazei, were less abundant in controls, but both appear to be artefactual detections. Analysis of reads aligning to Alteromonas mediterranea showed that the 5050 classified reads aligned to only 0.221% of the 4.6 million base pair genome, and some of these reads aligned to human DNA as well as the bacterial genome. Methanosarcina mazei also showed sparse coverage of the genome, with 1159 reads aligning to only 0.0098% of the 4.1 million base pair genome, and the sequences that mapped were of low complexity (Additional file 1: Table S13). Low-complexity sequences are known to be problematic in quality control and classification steps, and in some cases, these low-complexity samples aligned to human DNA as well as the bacterial genomes, likely explaining their presence.

A few placenta samples were also higher in Vibrio bacteria than were negative controls. We used DNA from Vibrio campbellii, a marine bacteria, as a positive control during sequence acquisition. Reads matching Vibrio campbellii formed over 2% of nonhuman placental reads and up to 31% of a single sample. We thus infer that barcode misreading during sequence acquisition [17] accounted for the detection of this organism.

We next asked whether any distinctions could be found among the different types of placenta samples. When we extracted lineages unique to placenta after excluding those found in negative controls (Additional file 1: Table S14), we recovered very few reads. The most abundantly represented taxon, Acidiplasma cupricumulans, was identified with only 12 reads in 11 samples total. Acidiplasma cupricumulans is an acidophile from the phylum Archaea and not a likely candidate for a placental colonist. Other organisms showed even lower read numbers, leaving us doubtful that any represented authentic placental colonists.

After filtering out probable artifacts and barcode misattributions (Alteromonas mediterranea, Methanosarcina mazei, and Vibrio campbellii), comparison of preterm and term deliveries showed a significant difference (*p* = 0.002, PERMANOVA, Jaccard distance). However, none of the taxa identified show more than 24 reads aligning to the target genome. (Additional file 1: Tables S15 and S16). The most abundant organism identified, Kytococcus sedentarius, was identified largely in a single preterm, fetal side placenta sample (20 out of 24 classified reads) that was from a pregnancy with preterm premature rupture of membranes (PPROM) and chorioamnionitis. Kytococcus sedentarius was discovered as a marine bacteria and is suggested to also be an opportunistic human pathogen [35]. Reads classified as Kytococcus sedentarius were also found in other samples including Saliva, Blanks, and H2O at similar or higher levels, suggesting this bacteria could be derived from reagent contamination. We thus find that all differential detections are of extremely low abundance and are sample specific. Comparison of vaginal deliveries and cesarean deliveries showed no distinction (*p* = 0.204, PERMANOVA, Jaccard distance). Comparison of fetal versus maternal side placenta samples showed no significant distinction (*p* = 0.169, PERMANOVA, Jaccard distance). Comparison of placenta samples from cases of chorioamnionitis to other placenta samples also showed no differences (Additional file 2: Figure S6). Thus, we conclude that analysis by shotgun metagenomic sequencing also did not disclose a detectable placenta microbiome, and consistent with the lack of signal, we did not see convincing biological distinctions in the data.

## Discussion

The existence of a placenta microbiome in healthy mothers remains controversial. Previously, we reported that we could not distinguish the putative microbiome in term placenta samples from the contamination background in negative controls [15]. Here, we repeated the study but compared samples from term and spontaneous preterm births, and also compared results obtained by 16S rRNA marker gene sequencing and shotgun metagenomic sequencing. Using 16S rRNA marker gene sequencing, we found that placenta samples were generally similar to background, although we did find low-level representation of vaginal lineages selectively in placentas from vaginal deliveries, suggestive of contamination of placenta samples with vaginal fluids. With shotgun metagenomic sequencing, the great majority of the sequences were human or contaminants shared with negative controls. Of the small number of nonhuman reads that differed between placenta and controls, most were low-complexity DNA sequences likely derived from human repeated sequences. We were unable to distinguish the placenta microbiome from background in either term and preterm births, though reaching this conclusion required filtering several forms of artifacts associated with low-biomass samples containing high amounts of human DNA. These data do not support the hypothesis that the placenta is normally colonized by a consistent bacterial community.

We were unable to detect any distinctive bacterial signature in placentas from cases of chorioamnionitis, which involves inflammation of the fetal membranes associated with bacterial infection. In a minority of cases, Ureaplasma and Mycoplasma could be detected in the 16S rRNA gene sequence data from preterm samples. However, these organisms were also usually detectable in vaginal swab samples from the same women, leaving it unclear whether these sequences originated from the placenta specimen or vaginal contamination during delivery. In favor of the contamination argument, Lactobacillus amplicon sequence variants characteristic of those inhabiting the vagina were similarly abundant in many samples from vaginal deliveries. However, it is possible that Ureaplasma and Mycoplasma are genuinely present in vagina and placenta in some of the pre-term birth samples. We did find one case where Ureaplasma was detected in a preterm birth sample associated with chorioamnionitis but not in vagina, possibly representing authentic detection of a pathogen in placenta. Thus, we feel our data may be consistent with 16S rRNA amplicon sequence results of Leon et al. [30], suggesting association of Ureaplasma and Mycoplasma in placenta from some preterm birth samples.

With this study and two previous failures to detect a normal placenta microbiome in samples from healthy term deliveries [15, 30], we question whether any bacterial community normally exists in this tissue and suggest that it is usually sterile until rupture of membranes during delivery. Thus, our studies on placenta are more broadly consistent with the sterile womb hypothesis. Infection may be detectable in a minority of placenta samples from pre-term birth, though here too possible contamination of placenta specimens with vaginal lineages provides an alternative explanation for most cases. Our data is congruent with the observation that axenic (germ free) mammals can be generated routinely [19, 20]—in numerous studies, mammalian neonates have been delivered by sterile cesarean section into sterile isolators, after which they remain sterile. It is hard to sustain the idea that the womb is normally colonized by microbes given this observation. Furthermore, two recent studies using deep DNA sequencing methods could not detect bacteria in the amniotic fluid of women with healthy pregnancies [36, 37]. In our view, available data is inconsistent with the colonized womb hypothesis and more consistent with the sterile womb hypothesis.

## Conclusions

We assayed samples from preterm and term deliveries using 16S rRNA gene sequencing and shotgun metagenomics. The expected microbial communities were found in oral and vaginal samples, but placenta samples could not be distinguished from the contamination background.

## Methods

### Human subjects

Subjects in this study were selected from a large case-control study (Cellular Injury and Preterm Birth, CRIB) funded by the March of Dimes Prematurity Research Center at the University of Pennsylvania. All enrollment details and sample collection methods can be found in [15] with the following addition. This study used samples from 20 subjects in the control group as well as 20 subjects identified as “cases.” The control group consisted of uncomplicated singleton pregnancies delivered at term following spontaneous labor (regular contractions, cervical dilation) or spontaneous rupture of membranes. Cases were defined as adult women with singleton pregnancies who delivered between 23–0/7 and 34–6/7 weeks gestational age following idiopathic preterm labor (regular contractions and cervical dilation), preterm premature rupture of membranes (PPROM), or cervical insufficiency (painless cervical dilation). The CRIB study has been approved by the Institutional Review Board at the University of Pennsylvania (protocol #821376).

### DNA purification

Two DNA extractions kits were used for this study, DNeasy PowerSoil (QIAGEN, Hilden, Germany) and QIAamp UCP Pathogen Mini (QIAGEN, Hilden, Germany).

PowerSoil kit: DNA was extracted from replicate maternal-side and fetal-side placental biopsies, mother’s saliva, air swabs, and reagent blanks. Whole placenta biopsies were placed directly into tared bead tubes, and ranged from 0.03 to 0.68 g. Between 33 and 100 μl of saliva was transferred into the tubes. Swabs were cut directly into the bead tubes. Samples were extracted as previously described [15]. Extracted DNA was stored at -20 °C.

UltraClean kit: DNA was extracted from replicate maternal-side and fetal-side placental biopsies, mother’s saliva, vaginal swabs, air swabs, and reagent blanks. For the vaginal swabs, air swabs, and saliva, samples were extracted as per the manufacturer’s protocol with the Mechanical Pre-lysis steps, using Pathogen Lysis Tubes S (QIAGEN, Hilden, Germany). Swabs were cut directly into microcentrifuge tubes. Between 30 and 100 μl of saliva was used for the extractions.

The optimum mass of a tissue sample for this extraction method is 0.25 g. In order to minimize the chance of contamination of the placenta tissues, whole samples were directly transferred into tared lysis tubes, and the mass was recorded. The tissue samples weighed between 0.06 and 0.45 g. Five hundred microliters of reagent ATL was added, and the samples were placed in a TissueLyser II (QIAGEN, Hilden, Germany) for 10 min. Forty microliters of proteinase K was added to each sample, and then they were incubated overnight at 56 °C. The samples were then placed in the TissueLyser again for 10 min. If the initial mass of the sample was greater than 0.25 g, a proportion of the supernatant, normalized to 0.25 g, was removed and diluted to 440 μl, and then extracted per manufacturer’s protocol. Extracted DNA was stored at − 20 °C.

### 16S rRNA gene quantitative PCR

Bacterial abundance was quantified using qPCR of the V1-V2 region of the 16S rRNA gene using a TaqMan-based assay (Applied Biosystems, Foster City, CA). Samples were run in triplicate. Primer and probe sequences are described in [38, 39] and are presented in Additional file 1: Table S17. Reactions were analyzed on a QuantStudio 5 Realtime PCR System (Thermo Fisher Scientific, Waltham, MA).

### 16S rRNA marker gene amplification and sequencing

The V1-V2 regions of the 16S rRNA gene was amplified in each sample using Golay-barcoded primers 27F and 338R [40, 41]. Samples were amplified in quadruplicate, then pooled and bead purified using Agencourt AMPure XP beads (Beckman Coulter, Brea, CA). Amplified products were sequenced on the Illumina MiSeq platform (Illumina, San Diego, CA).

### Analytical methods used for 16S rRNA marker gene data

Sequence data was processed using DADA2 [42]. Reads were trimmed from 251 bases to 240. Dereplication, error modeling, denoising, pair merging, and chimera removal were performed using default parameters. Taxonomic assignments were generated by comparison to the Silva reference database [43–45]. Samples with fewer than 100 reads were excluded from downstream analysis. Beta diversity analysis (unweighted UniFrac and weighted UniFrac) was performed using the beta_diversity.py script from Qiime 1.9.1 [46]. Jaccard, PCoA, and PERMANOVA analysis were performed using functions vegdist, pcoa, and adonis from the R package “vegan” [47].

### Shotgun metagenomic sequencing

Samples were prepared for metagenomic sequencing with the Nextera XT DNA Library Prep Kit and sequenced on the Illumina HiSeq 2500 (Illumina, San Diego, CA) with dual-indexed barcodes and 2 × 125 base read lengths.

### Analytical methods used for shotgun sequence data

The shotgun metagenomic sequence reads were processed using the Sunbeam pipeline [32]. Reads were quality controlled with Trimmomatic [48], to remove low-quality bases and adapter sequences. Host reads were then identified using BWA [49] and removed. The remaining reads were taxonomically classified using Kraken [33] with a custom database built on all bacterial, fungal, archaeal, and viral genomes in RefSeq release 79 [50]. To further filter our data of problematic, low-complexity reads, we used Komplexity to remove low-complexity reads that remained after host decontamination [32]. As a final filtering step, we excluded reads that were classified by Kraken as Chordata, Arthropoda, and Apicomplexa. Sequencing reads were aligned to target genomes using BWA-MEM [49], and genome coverage was calculated using BEDtools [51].

## Additional files

Additional file 1: **Table S1.** Human subjects studied. AA indicates African American, SVD is spontaneous vaginal delivery, (P)PROM is (preterm) premature rupture of the membranes. **Table S2.** Description of sample types. **Table S3.** Summary of samples analyzed by 16S rRNA marker gene sequencing and shotgun metagenomic sequencing. **Table S4.** Statistical analysis of 16S rRNA gene qPCR data. CT values were compared using the Kruskal-Wallis Test with Dunn’s Post-test. The comparison between the placenta samples and Air Swabs show a significant difference (*p* < 0.05), however, the Air Swab samples had lower CT values than placenta samples, and therefore higher bacterial counts. **Table S5.** Statistical analysis of 16S rRNA gene qPCR data of the term birth groups. CT values were compared using the Kruskal-Wallis Test with Dunn’s Post-test. **Table S6.** Statistical analysis of 16S rRNA gene qPCR data of the preterm birth groups. CT values were compared using the Kruskal-Wallis Test with Dunn’s Post-test. **Table S7.** Statistical analysis of 16S rRNA gene qPCR data of the birth groups diagnosed with chorioamnionitis. CT values were compared using the Kruskal-Wallis Test with Dunn’s Post-test. **Table S8.** Statistical analysis of 16S rRNA gene qPCR data of the birth groups not diagnosed with chorioamnionitis. CT values were compared using the Kruskal-Wallis Test with Dunn’s Post-test. **Table S9.** Statistical analysis of 16S rRNA gene qPCR data of the samples purified with the PowerSoil extraction kit. CT values were compared using the Kruskal-Wallis Test with Dunn’s Post-test. **Table S10.** Statistical analysis of 16S rRNA gene qPCR data of the samples purified with the UltraClean extraction kit. CT values were compared using the Kruskal-Wallis Test with Dunn’s Post-test. **Table S11.** Read counts for sequences acquired for each sample in the 16S rRNA marker gene sequencing and shotgun sequencing studies. **Table S12.** *P*-values for comparisons among groups as analyzed using Adonis permutation tests applied to the 16S rRNA marker gene sequencing data. All values were corrected for multiple comparisons. The comparisons tested are indicated above the columns; the samples tested are in the rows. **Table S13.** Sequences from metagenomic analysis of placenta samples, showing the most abundant sequences aligning to Methanosarcina mazei and Alteromonas mediterranea. **Table S14.** Kraken classified taxa that are found in placenta samples after removing those that are found in the negative controls. The counts are the sums of classified reads within the group. Only taxa with at least two classified reads are shown. **Table S15.** Kraken classified taxa that are unique to placentas from term births and absent from placentas from preterm births (reads found in negative controls were not removed). The counts are the sums of classified reads within the group. Only taxa with at least two classified reads are shown. **Table S16.** Kraken classified taxa that are unique to placentas from preterm births and absent from placentas from term births (reads found in negative controls were not removed). Only taxa with at least two classified reads are shown. **Table S17.** Oligonucleotides used in this study. (XLS 158 kb)

Additional file 2: **Figure S1.** Numbers of sequence reads from the 16S rRNA marker gene analysis classified by Dada2. **Figure S2.** Heat map representation of bacterial lineages in each sample reported by 16S rRNA marker gene sequencing. The figure is the same as Fig. 2a, but shows the lowest level phylogenetic attribution (left column). **Figure S3.** Heat map of taxa seen in placenta samples using 16S rRNA marker gene sequencing, with easier viewing of the comparison between different sample types. **Figure S4.** Heat map of amplicon sequence variants (ASVs) seen in placenta and vaginal swab samples using 16S rRNA marker gene sequencing grouped by patient. “SVD” is standard vaginal delivery and “chorioam.” is chorioamnionitis. ASVs present had at least 25 reads for one sample. **Figure S5.** Heat map representation of microbial lineages in each sample reported by shotgun metagenomic sequencing. The figure is the same as Fig. 4, but shows the lowest level phylogenetic attribution (left column). **Figure S6.** Heat map of taxa detected in placenta samples using shotgun metagenomic sequencing, with easier viewing of the comparison between sample types. (PDF 2181 kb)
