# Supplementary figures and images for: Lack of detection of a human placenta microbiome in samples from preterm and term deliveries
Source: Microbiome. 2018 Oct 30;6:196. doi: 10.1186/s40168-018-0575-4 (PMC6208038; doi:10.1186/s40168-018-0575-4)

# Figure S1

16s rRNA Gene Sequence Reads

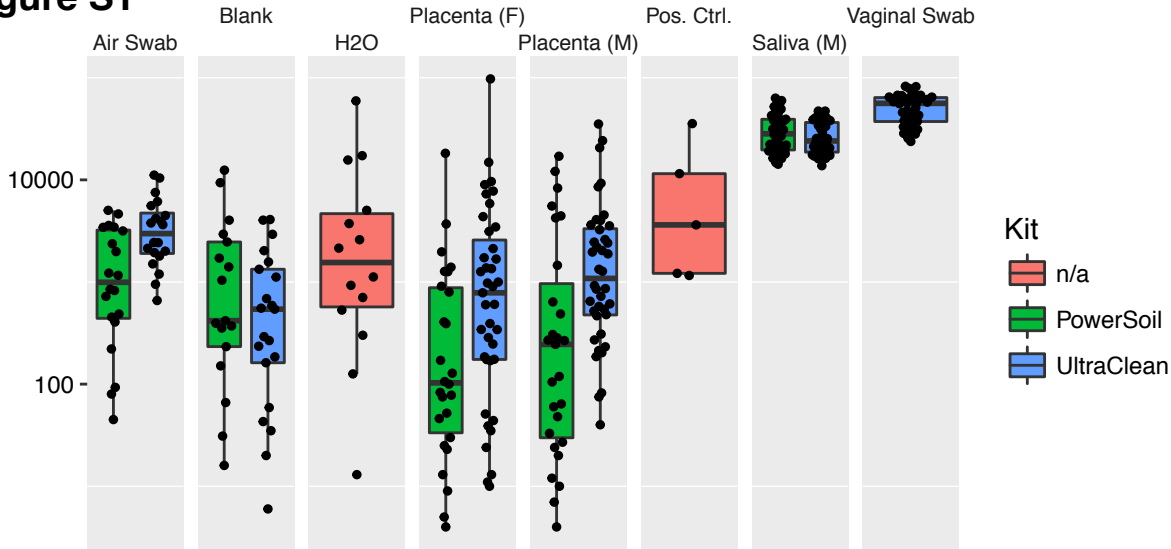

Figure S2

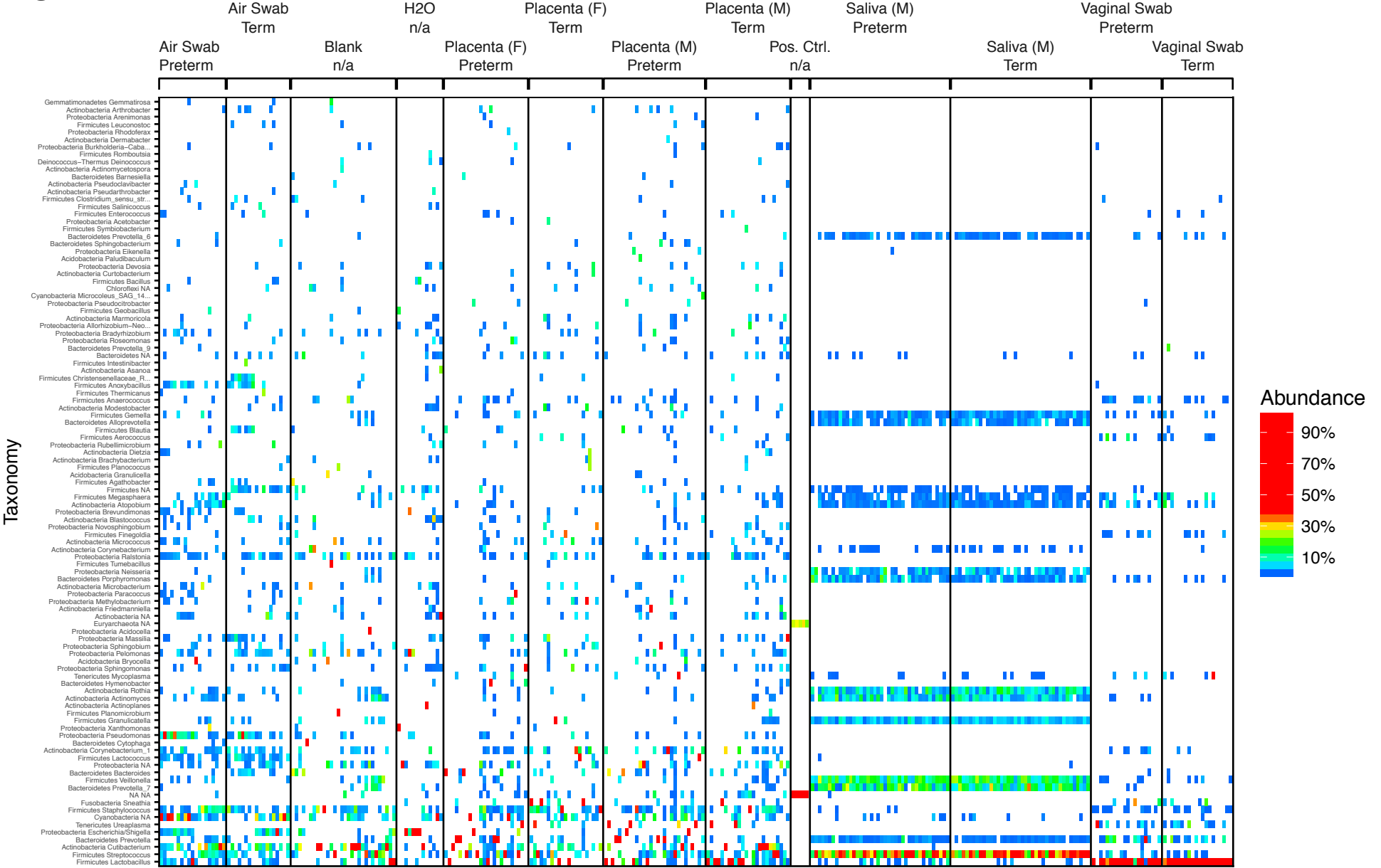

Figure S3

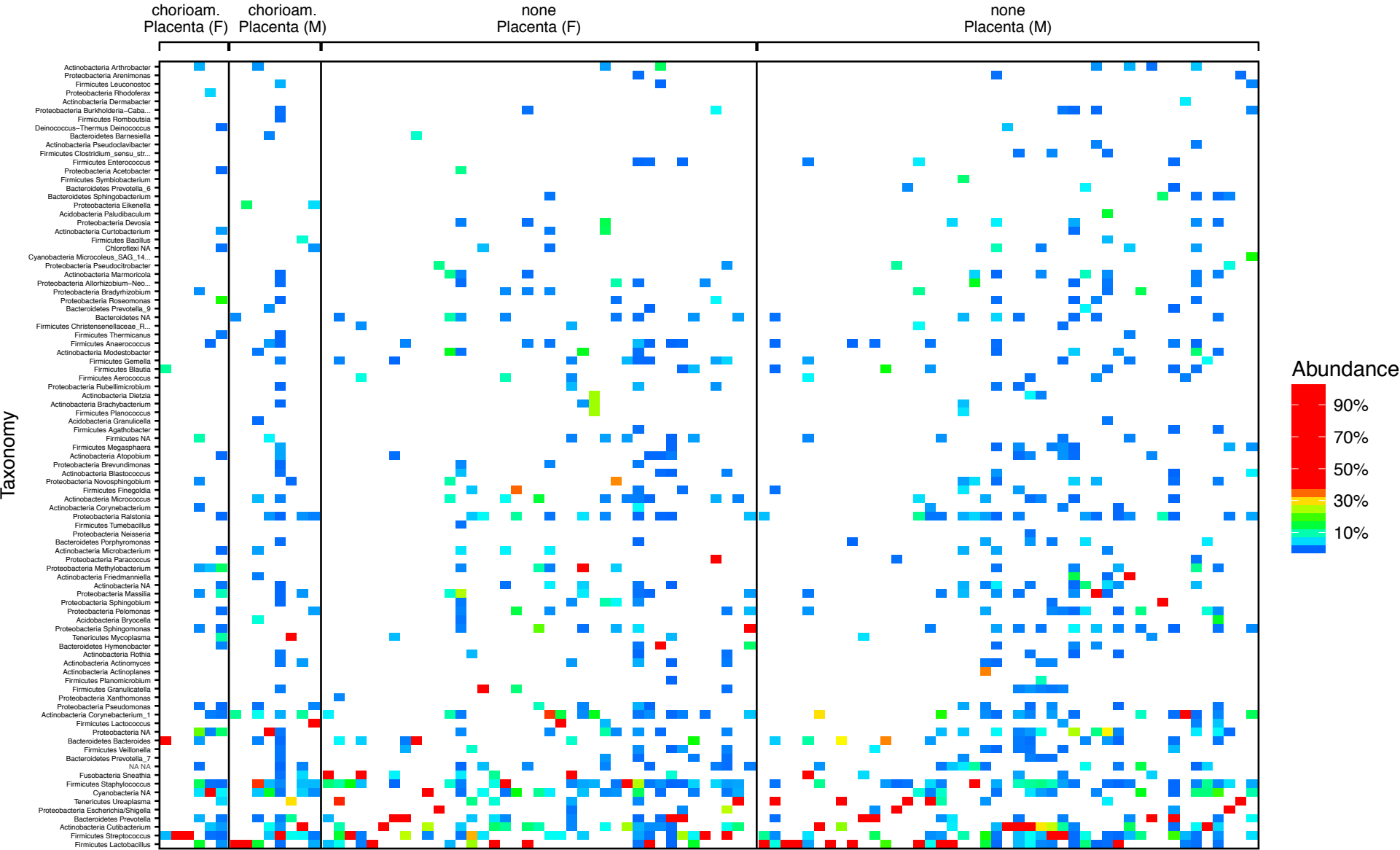

Figure S4

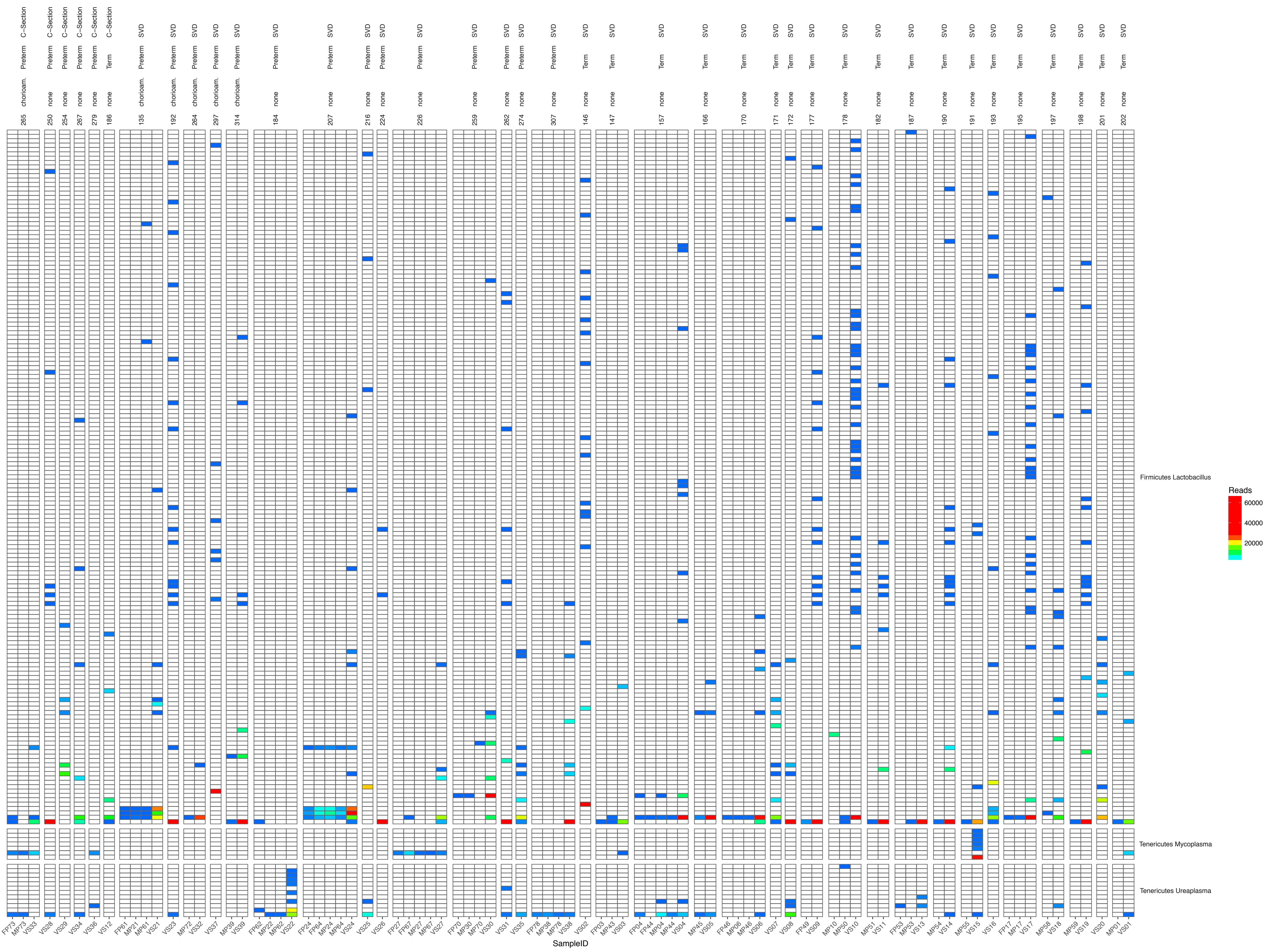

Figure S5

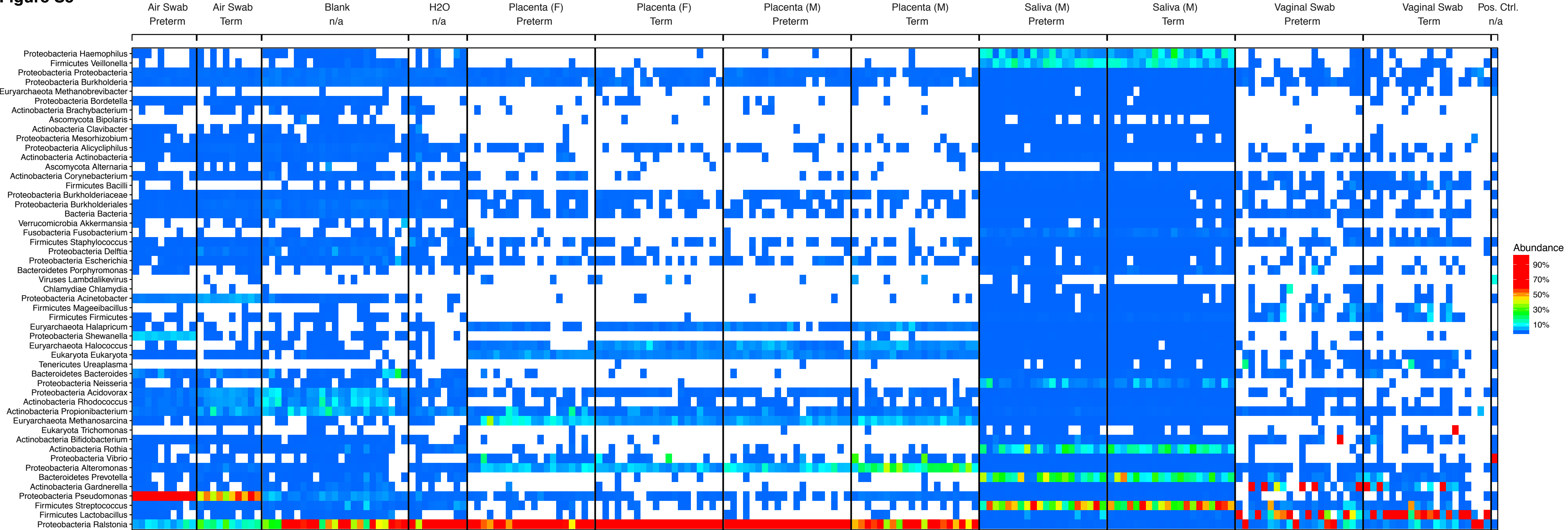

Figure S6

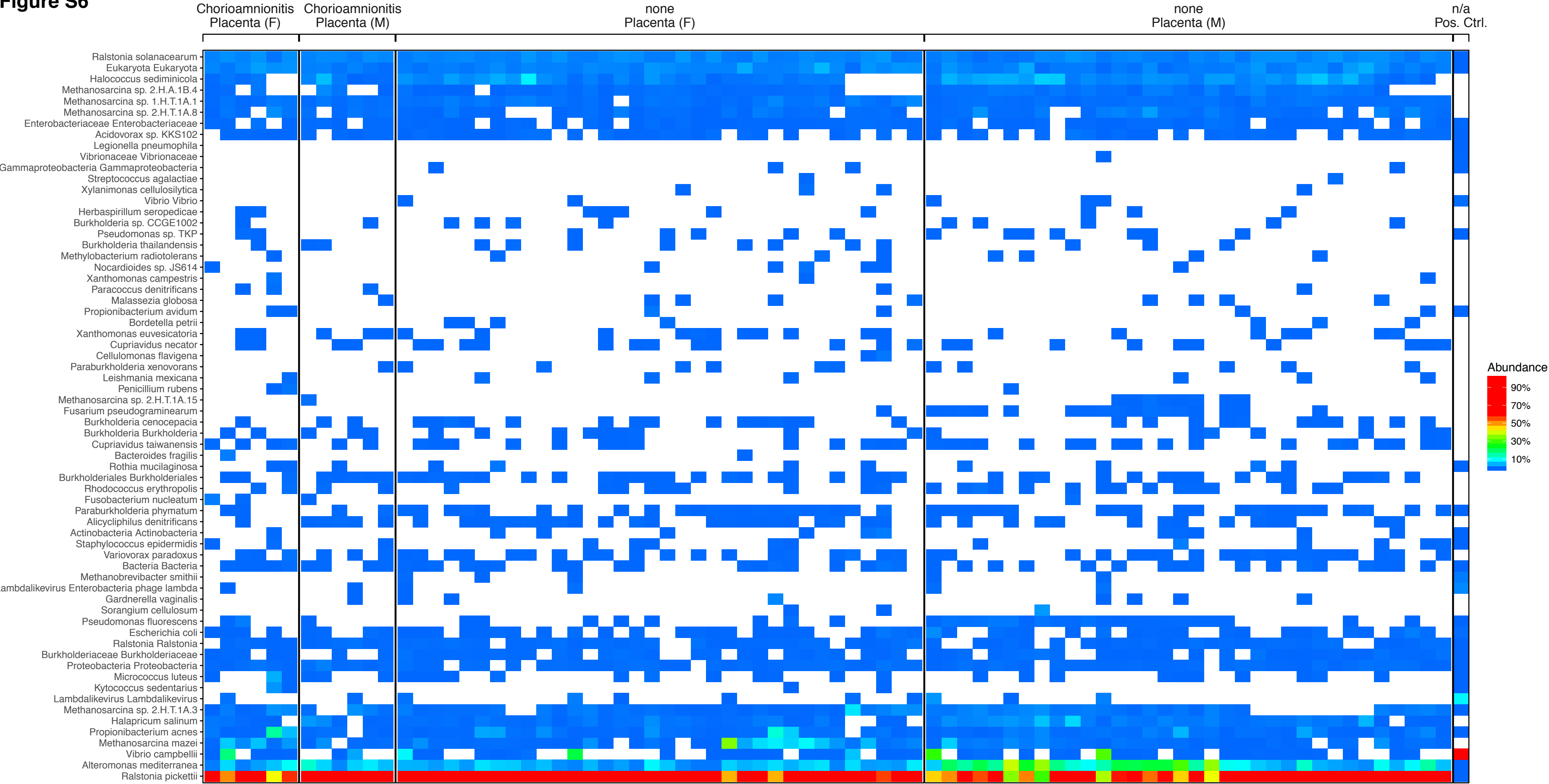

Supplement: Supplementary file 2 — Figure S1. Numbers of sequence reads from the 16S rRNA marker gene analysis classified by Dada2. Figure S2. Heat map representation of bacterial lineages in each sample reported by 16S rRNA marker gene sequencing. The figure is the same as Fig. 2a, but shows the lowest level phylogenetic attribution (left column). Figure S3. Heat map of taxa seen in placenta samples using 16S rRNA marker gene sequencing, with easier viewing of the comparison between different sample types. Figure S4. Heat map of amplicon sequence variants (ASVs) seen in placenta and vaginal swab samples using 16S rRNA marker gene sequencing grouped by patient. “SVD” is standard vaginal delivery and “chorioam.” is chorioamnionitis. ASVs present had at least 25 reads for one sample. Figure S5. Heat map representation of microbial lineages in each sample reported by shotgun metagenomic sequencing. The figure is the same as Fig. 4, but shows the lowest level phylogenetic attribution (left column). Figure S6. Heat map of taxa detected in placenta samples using shotgun metagenomic sequencing, with easier viewing of the comparison between sample types. (PDF 2181 kb) [file 40168_2018_575_MOESM2_ESM.pdf]
